# Targeting homologous recombination deficiency in uterine leiomyosarcoma

**DOI:** 10.1186/s13046-023-02687-0

**Published:** 2023-05-04

**Authors:** Genevieve Dall, Cassandra J. Vandenberg, Ksenija Nesic, Gayanie Ratnayake, Wenying Zhu, Joseph H. A. Vissers, Justin Bedő, Jocelyn Penington, Matthew J. Wakefield, Damien Kee, Amandine Carmagnac, Ratana Lim, Kristy Shield-Artin, Briony Milesi, Amanda Lobley, Elizabeth L. Kyran, Emily O’Grady, Joshua Tram, Warren Zhou, Devindee Nugawela, Kym Pham Stewart, Reece Caldwell, Lia Papadopoulos, Ashley P. Ng, Alexander Dobrovic, Stephen B. Fox, Orla McNally, Jeremy D. Power, Tarek Meniawy, Teng Han Tan, Ian M. Collins, Oliver Klein, Stephen Barnett, Inger Olesen, Anne Hamilton, Oliver Hofmann, Sean Grimmond, Anthony T. Papenfuss, Clare L. Scott, Holly E. Barker

**Affiliations:** 1 Walter and Eliza Hall Institute of Medical Research, Parkville, VIC 3052 Australia; 2 Department of Medical Biology, University of Melbourne, Parkville, VIC 3052 Australia; 3 Royal Women’s Hospital, Parkville, VIC 3052 Australia; 4 Centre for Cancer Research and Department of Clinical Pathology, University of Melbourne, Parkville, VIC 3010 Australia; 5 School of Computing and Information Systems, the University of Melbourne, Parkville, VIC 3010 Australia; 6 Olivia Newton-John Cancer Research Institute, Heidelberg, VIC 3084 Australia; 7 Austin Health, Heidelberg, VIC 3084 Australia; 8 Australian Rare Cancer Portal, BioGrid Australia, Melbourne Health, Parkville, VIC 3052 Australia; 9 Peter MacCallum Cancer Centre and Sir Peter MacCallum Department of Oncology, The University of Melbourne, Victoria, 3010 Australia; 10 Royal Melbourne Hospital, Parkville, VIC 3052 Australia; 11 Department of Obstetrics and Gynaecology, University of Melbourne, Parkville, VIC 3010 Australia; 12 Launceston General Hospital, Launceston, TAS 7250 Australia; 13 University of Western Australia, Perth, WA 6009 Australia; 14 SouthWest Healthcare, Warrnambool, VIC 3280 Australia; 15 Faculty of Health, School of Medicine, Deakin University, Warrnambool, VIC 3280 Australia; 16 Western Hospital, Footscray, VIC 3011 Australia; 17 University Hospital Geelong, Geelong, VIC 3220 Australia

**Keywords:** Uterine leiomyosarcoma, Homologous recombination deficiency, PARP inhibitors, Rare cancers, Patient-derived xenografts

## Abstract

**Background:**

Uterine leiomyosarcoma (uLMS) is a rare and aggressive gynaecological malignancy, with individuals with advanced uLMS having a five-year survival of < 10%. Mutations in the homologous recombination (HR) DNA repair pathway have been observed in ~ 10% of uLMS cases, with reports of some individuals benefiting from poly (ADP-ribose) polymerase (PARP) inhibitor (PARPi) therapy, which targets this DNA repair defect. In this report, we screened individuals with uLMS, accrued nationally, for mutations in the HR repair pathway and explored new approaches to therapeutic targeting.

**Methods:**

A cohort of 58 individuals with uLMS were screened for HR Deficiency (HRD) using whole genome sequencing (WGS), whole exome sequencing (WES) or NGS panel testing. Individuals identified to have HRD uLMS were offered PARPi therapy and clinical outcome details collected. Patient-derived xenografts (PDX) were generated for therapeutic targeting.

**Results:**

All 13 uLMS samples analysed by WGS had a dominant COSMIC mutational signature 3; 11 of these had high genome-wide loss of heterozygosity (LOH) (> 0.2) but only two samples had a CHORD score > 50%, one of which had a homozygous pathogenic alteration in an HR gene (deletion in *BRCA2*). A further three samples harboured homozygous HRD alterations (all deletions in *BRCA2),* detected by WES or panel sequencing, with 5/58 (9%) individuals having HRD uLMS. All five individuals gained access to PARPi therapy. Two of three individuals with mature clinical follow up achieved a complete response or durable partial response (PR) with the subsequent addition of platinum to PARPi upon minor progression during initial PR on PARPi. Corresponding PDX responses were most rapid, complete and sustained with the PARP1-specific PARPi, AZD5305, compared with either olaparib alone or olaparib plus cisplatin, even in a paired sample of a *BRCA2*-deleted PDX, derived following PARPi therapy in the patient, which had developed PARPi-resistance mutations in *PRKDC*, encoding DNA-PKcs.

**Conclusions:**

Our work demonstrates the value of identifying HRD for therapeutic targeting by PARPi and platinum in individuals with the aggressive rare malignancy, uLMS and suggests that individuals with HRD uLMS should be included in trials of PARP1-specific PARPi.

**Supplementary Information:**

The online version contains supplementary material available at 10.1186/s13046-023-02687-0.

## Background

Uterine leiomyosarcoma (uLMS) is a malignant tumour of the myometrium and accounts for approximately 60% of uterine sarcomas [1]. Despite 5-year survival rates of 42–76% for individuals presenting with early-stage disease [2, 3], recurrence is common [4], and metastatic disease is often present at diagnosis. As a result, uLMS accounts for almost 70% of uterine sarcoma deaths [5]. While early-stage disease is usually treated with hysterectomy, with or without bilateral salpingo-oophorectomy, chemotherapy is reserved for metastatic or recurrent disease [5, 6], but despite this, the 5-year survival rate for individuals with advanced uLMS is < 10%. Radiotherapy is often used as adjuvant therapy in early-stage disease in order to reduce local recurrence, however it does not appear to impact overall survival [7, 8].

Molecular analyses using WES [9–14], targeted panel sequencing [15–17] and limited WGS [11, 14] have revealed the most commonly altered genes in uLMS to be *TP53* followed by *RB1*, *ATRX*, *PTEN* and *MED12*. WGS revealed that a high proportion of tumours (76%) harbour chromothripsis/chromoplexy [14]. One study, identified frequent focal amplification of chromosome regions containing the genes *TERT*, *MAP2K4*, *MYOCD* and *C-MYC,* and frequent focal deletions of regions containing the genes *RB1*, *TP53*, *PTEN*, *CDKN2A*, *CYLD* and *BRCA2* [14]. RNA sequencing has also identified frequent fusion genes disrupting multiple tumour suppressor genes, such as RB1, TP53, ATRX, DAXX, CAMTA1, SETD2 and KDM5CA [14]. Currently, none of these aberrations are routinely targeted therapeutically in individuals with uLMS [18] and thus treatment strategies continue unchanged and disease outcomes have remained stagnant for decades.

In ovarian cancer, significant advances in clinical efficacy have been attributed to the use of PARPi in predominantly high-grade epithelial serous ovarian, fallopian tube or primary peritoneal carcinomas (HGSOC) displaying homologous recombination deficiency (HRD) in DNA damage repair [19–22]. In a recent meta-analysis of four trials of platinum-sensitive relapsed HGSOC (including Study 19, SOLO2, ARIEL3 and NOVA) encompassing 972 patients, maintenance PARPi improved progression free survival (PFS) compared to placebo regardless of whether the *BRCA1/2* mutation was germline or somatic (p = 0.48) and HRD HGSOC had better outcomes than did HR proficient cases (*p* < 0.00001) [23]. Several case reports demonstrating the clinical efficacy of PARPi in endometrial/uterine carcinoma cases have been published in recent years [24–26] leading to the establishment of clinical trials investigating the efficacy of the PARPi, niraparib, rucaparib or olaparib, in advanced / metastatic endometrial cancer (NCT03016338, NCT03617679, NCT04269200, NCT03981796).

Unlike individuals with other gynaecological malignancies, who are typically referred to familial genetics clinics for germline *BRCA1/2* testing, individuals with uLMS are not routinely screened for such germline mutations in the clinic, although some individuals with uLMS have been shown to carry germline mutations in *TP53* or *RB1* [18], albeit at a low rate (below the internationally accepted cut off of ~ 10% prevalence for germline mutations in the target population requiring testing [27]). In their reanalysis of the TCGA and GENIE soft-tissue sarcoma datasets, in addition to their own uLMS cohort, Seligson and associates observed that alterations in *BRCA1/2*, concluded to be somatic, were significantly more common in uLMS compared with non-uterine LMS (10% compared to 1%, respectively, *p*-value 0.02, *n* = 61 patients) [28]. In that study, other less well substantiated genes in the HR pathway were found to be mutated in an additional 13% of cases of uLMS and alterations in the HR pathway have been reported at a higher frequency in cases of uLMS compared with non-uterine LMS [29]. In addition, Choi and colleagues identified COSMIC mutational signature 3, which is proposed to correlate with HRD, as the dominant signature in 25% of uLMS tumours [14]. PARPi therapy has been shown to have utility in pre-clinical models of uLMS [10, 14] as well as in individuals with uLMS [15, 28]. Indeed, by way of emphasising the importance of the HR pathway, striking efficacy has been observed with single agent PARPi even in individuals with advanced uLMS, with individuals with alterations in *BRCA2* in their uLMS achieving partial response (PR) or stable disease (SD), documented for 15 months or longer [15, 28], with one complete response (CR) having been reported [15].

Through the WEHI-Stafford Fox Rare Cancer Program [30], 51 Australian individuals with uLMS were screened for alterations in genes in the HR pathway using either panel testing, WES or WGS. We detected six pathogenic aberrations in *BRCA2* in uLMS, four deletions (all homozygous) and two mutations (both heterozygous), all somatic. We also detected a number of cases with dominant COSMIC mutational signature 3, which is known to correlate with HRD [31] and with sensitivity to PARPi in cancer cell lines [32]. One of these cases had a copy number (CN) profile consistent with LOH in the PDX, which was of high tumour purity, however HRDetect and CHORD scores were not available due to low tumour purity of the patient sample (HRDetect defines a score greater than 0.7 as HRD [33], and CHORD defines a percentage of > 50% as HRD [34]). The rate of HRD aberrations was therefore five out of 58 cases (8.6%). Here, we report on five individuals who received PARPi as part of their treatment for HRD uLMS, two of whom achieved either a CR or ongoing PR after platinum was added to olaparib, upon progression of disease during single-agent olaparib therapy. We have validated the relative responsiveness of HRD uLMS to PARPi using HRD and HR proficient (HRP) patient-derived xenografts (PDX). When compared with olaparib treatment, we demonstrated superior efficacy for an HRD uLMS PDX treated with the combination of olaparib with cisplatin, with the best response observed with the PARP1-specific PARPi, AZD5305 as a single agent, even in a paired PDX containing PARPi-resistance mutations. Herein, we provide additional evidence to support clinical screening for HRD in individuals with uLMS, in order to support their access to PARPi treatment regimens, which can be transformational for some individuals.

## Materials and methods

### Clinical samples

Tumour, blood samples and clinical data were obtained from patients enrolled in the WEHI-Stafford Fox Rare Cancer Program (SFRCP), approved by Melbourne Health Human Research Ethics Committee (HREC) (2015.300), which recruits Australia-wide, including a remote consent option. Informed consent was obtained from all patients in accordance with the National Statement of Ethical Conduct in Human Research 2007. Additional approval was obtained from the Human Research Ethics Committee at the Peter MacCallum Cancer Centre. Cancer specialists throughout Australia were contacted via the Australia New Zealand Gynaecological Oncology Group (ANZGOG) (https://www.anzgog.org.au/) and the Australian Rare Cancer (ARC) Portal (https://www.arcportal.org.au/) and encouraged to refer their patients for molecular screening. Flyers were approved (Melbourne Health HREC) for patient use, to inform patients and their doctors about the SFRCP and ARC-Portal programs, thus empowering patient involvement in this research. In order to reduce the chance of a person with a rare cancer being identified, only hemi-decile age is provided to researchers. For the two cases described in detail (*BRCA2* deleted, receiving PARPi/platinum combination therapy), an additional Patient Information Consent Form was signed by each patient, providing permission to publish the clinical details of their case, with the understanding that such details may result in their case being identifiable (Approved by MH HREC 2015.300).

### Histology

Formalin fixed paraffin-embedded (FFPE) tumour samples were sectioned and stained with haematoxylin and eosin (H&E) before pathological review and determination of tumour purity. Sections were also stained with anti-smooth muscle actin (Clone E184, Abcam), anti-desmin (Polyclonal, Abcam), anti-Ki67 (MIB-1, Dako), and anti-PAX8 (polyclonal, Proteintech) using the Ventana BenchMark Ultra fully automated staining instrument (Roche Diagnostics, USA). H&E and IHC slides were digitally scanned (20 × magnification) using the Pannoramic 1000 scanner (3DHISTECH Ltd.). High-definition images were uploaded into CaseCenter (3DHISTECH Ltd.), and images were processed using Adobe Illustrator.

### Whole genome sequencing

WGS of patient samples was performed on DNA extracted from fresh frozen tissue and matched blood. 200 ng of DNA was fragmented to approximately 550 bp using a focused-ultrasonicator (Covaris M220). Libraries were prepared with the Illumina TruSeq nano DNA library preparation kit. The libraries were molecularly barcoded with IDT for Illumina TruSeq DNA Unique Dual Index adapters prior to pooling and sequencing to a depth of 40 × for the normal and 80 × or 100 × for tumour using paired 150 bp reads on the Illumina NovaSeq 6000 platform.

WGS of PDX tumours was performed on DNA extracted from fresh frozen tissue. Libraries were prepared using the Nextera Flex library method (Illumina). Indexed libraries were sequenced to a depth of 60 × using paired 150 bp reads on the Illumina Novaseq 6000 platform.

### Whole exome sequencing

WES of patient samples was performed on DNA extracted from FFPE tumour tissue and matched blood. 150–300 ng of DNA was fragmented to approximately 200 bp using a focal acoustic device (Covaris S2, Sage Sciences). Libraries were prepared with the Kapa Hyper Prep Kit (Kapa Biosystems) and SureSelectXT adaptors (Agilent). Hybridisation capture was performed with SureSelect Clinical Research Exome V2 baits following the SureSelectXT recommended protocol (Agilent). Indexed libraries were sequenced on an Illumina NovaSeq 6000 (Illumina) to generate on average 200 million paired-end 150 bp reads per sample.

### Analysis of sequencing data

Initial WGS analysis was performed using a pipeline developed in the University of Melbourne Centre for Cancer Research as follows (hereafter referred to as the UoM pipeline). Sequence reads were aligned to the hg38 build of the human reference genome using BWA mem. Variants were detected by at least 2 of the following mutation callers (Mutect2, Strelka2 & Vardict) using the BCBIO pipeline (https://github.com/chapmanb/bcbio-nextgen). All variants were annotated using the personalised cancer genome reporter (https://github.com/sigven/pcgr). Single Nucleotide Variants (SNV)/Indels were classified according to a five-tiered structure, similar to proposed recommendations [35], also adopting the MLVD framework for description of clinically relevant cancer variants. Tumour mutational burden (TMB) was defined as the number of coding, somatic substitutions and indels, including synonymous alterations, per megabase of the targeted coding genomic region (34 MB) [36]. Copy number variants were called using PURPLE [37]. Structural variants were detected using MANTA (https://github.com/illumina/manta) and BreakPointInspector [37]. CNV and SV changes are annotated with the *svprioritize* (https://github.com/AstraZeneca-NGS/simple_sv_annotation) framework assigning priority to fusion events, whole exon loss or upstream/downstream changes for a list of 1246 cancer-associated genes only (https://github.com/umccr/workflows/blob/master/genes/cancer_genes/umccr_cancer_genes.latest.ts). Somatic mutations are assigned to COSMIC v2 mutational signatures [38] using the MutationalPatterns framework [39]. HRD was detected by HRDetect [33] and CHORD [34]. HRDetect and CHORD consider mutational patterns (SNVs, Indels and Structural Variants) that are characteristic of HRD tumours. HRDetect grants a score from 0 to 1; tumour samples with a score > 0.7 are categorised as HRD. CHORD classifies tumours in BRCA1-deficient and BRCA2-deficient categories; tumours with a combined probability of < 50% HRD are categorised as HR-proficient.

WGS from PDX and WES of patient samples were analysed and all patient WGS were re-analysed using a bionix pipeline (https://github.com/PapenfussLab/bionix) [40] developed at WEHI as follows (hereafter referred to as the WEHI pipeline). All sequencing reads were aligned to the GRCh38p31 build [41] of the human reference genome. Reads from PDX were also aligned to the mm10p4 build of the mouse reference genome using minimap2 (v2.24) [42]. For PDX, reads unambiguously mapping to the human reference genome were then extracted using Xenomapper for subsequent variant calling [43]. Octopus (v0.7.0) [44] was used to call and phase SNVs and indels with subsequent annotation against Ensembl (v99) [45]. For WES, Octopus (v0.7.0) was applied within regions ± 100 bp of exon boundaries. dbNSFP (v4.2a) [46] using SnpEff and SnpSift (v4.3t) [47]. Structural variants were called using GRIDSS (v2.13.2) [48, 49]. Mutations were assigned to COSMIC v2 mutational signatures [38] using MutationalPatterns (v3.4.0) [39]. Copy number variants were called using FACETS (0.6.1) [50]. Default parameters were used except for uLMS122 and uLMS227 (cval = 50 and snp.nbhd = 500 for preProcSample; and cval = 400 for procSample) and the low-purity sample uLMS147 (cval = 100 and snp.nbhd = 750 for preProcSample; and cval = 700 for procSample). The presence of whole genome doubling was inferred using wgd.test (https://github.com/PapenfussLab/wgd.test) [51].

### BRCA assay

Tumour BRCA testing was performed by the Pathology Laboratory, Peter MacCallum Cancer Centre. DNA extracted from FFPE tumour tissue was screened for all coding exons and flanking intron junctions of the *BRCA1* and *BRCA2* genes using the QIAGEN UMI based QIASeq Targeted DNA Panel (DHS-102Z) Next Generation sequencing (NGS) kit. Indexed libraries were pooled and sequenced with the Illumina MiSeq v2 kit (2 × 150 bp). The QIAGEN laboratory software Biomedical Genomics Workbench version 5.0.1 was used to annotate and transform variants to standard nomenclature and filter for rare, non-synonymous variants within 5 bp of coding exons. Variants are described according to HGVS nomenclature version 15.11 (http://varnomen.hgvs.org/) with minor differences in accordance with Molecular Pathology policy.

### TruSight Oncology 500 (TSO500) panel testing

Targeted sequence analysis was performed at Garvan Institute of Medical Research. DNA and RNA was extracted from FFPE tumour material using Qiagen AllPrep DNA/RNA FFPE kit, and libraries were created and enriched using the Illumina TruSight Oncology 500 reagents kit including DNA and RNA probes panel. Samples were uniquely indexed, pooled and sequenced on an Illumina NextSeq500 to generate 2 × 100 bp reads at a target coverage of approximately 1000 reads/base.

### DNA methylation by Methylation-Sensitive High-Resolution Melting (MS-HR)

Where DNA was available methylation patterns of *BRCA1* and *RAD51C* promoters were assessed by MS-HRM [52] on the Magnetic Induction Cycler (Bio Molecular Systems, Upper Coomera, Queensland) thermocycler platform. Primers targeting the *RAD51C* promoter across genomic region chr 17:56,769,849–56,769,990 (hg19) were used as previously described [53].

### PDX generation and treatments

All animal experiments were conducted according to the National Health and Medical Research Council Australian Code for the Care and Use of Animals for Scientific Purposes 8^th^ Edition, 2013 (updated 2021), and under the approval of the WEHI Animal Ethics Committee (2019.024). Tumour fragments (1–3mm^3^) were implanted subcutaneously or into the ovarian bursa of NOD-SCID-IL-2Rgamma (NSG) mice under anaesthesia. Tumour growth was monitored weekly and once tumours reached 700mm^3^ the mice were euthanised and tumours excised (T1). T1 tumour fragments were transplanted into recipient NSG mice (T2) for serial transplantation, snap frozen, fixed in formalin and viably frozen in 10% DMSO/39%FCS/1% pen strep/50%DMEM. The patient and PDX T1 tumours were assessed by an expert gynaecological pathologist, including using immunohistochemistry (IHC), to validate the identity of the PDX tumour.

Mice bearing T2–T9 tumours that had reached 180–300mm^3^ in size were randomly allocated to a treatment group: DPBS/vehicle, caelyx (liposomal doxorubicin), olaparib, cisplatin, and combination cisplatin plus olaparib. Cisplatin (Pfizer) diluted in DPBS was delivered at 4 mg/kg on days 1, 8 and 18 intraperitoneally. Olaparib (MedChemExpress) solubilised in DMSO and diluted in 10% 2-hydroxypropyl-β-cyclodextrin (Sigma) was administered at either 100 mg/kg or 150 mg/kg daily (Monday to Friday) for 3 weeks or 6 weeks intraperitoneally. Caelyx (liposomal doxorubicin) (Janssen-Cilag) diluted in DPBS was administered at 1.5 mg/kg intravenously weekly for three weeks. AZD5305 was prepared weekly in sterile water pH3.5–4 (pH adjusted with 1N HCl) with bath sonication, and administered by daily oral gavage for 28 days at either 1 or 10 mg/kg.

Tumours were measured twice weekly, and mice were euthanised at experimental endpoints of either tumour volume > 700mm^3^ or 120 days post treatment initiation. Data collection was conducted using the Studylog LIMS software (Studylog Systems, San Francisco). Graphing and statistical analysis (pairwise log rank tests) was conducted using the SurvivalVolume package [54].

## Results

### A subset of the uLMS cohort were found to be HR defective

uLMS from 58 individuals were screened for mutations in genes in the HR pathway using either targeted panel testing, WES or WGS. Clinical characteristics for each individual are shown in Table 1. The most common hemi-decile age at diagnosis was 50–54 (precise ages not reported to aid deidentification), and most patients had undergone a total abdominal hysterectomy (TAH). Approximately half of the cohort had metastatic disease at diagnosis and the average primary tumour size was greater or equal to 10 cm.

**Table 1:** uLMS cohort characteristics

**Patients**		**58**
**Age at Diagnosis**		
	** ≤ 39**	2
	**40–44**	7
	**45–49**	12
	**50–54**	14
	**55–59**	8
	**60–64**	5
	**65–69**	2
	**70 + **	5
	**Unknown**	3
**FIGO stage**		
	**I**	10
	**II**	3
	**III**	2
	**IV**	8
	**Unknown**	35
**TAH**		
	**Yes**	51
	**No**	2
	**Unknown**	5
**Metastatic at Diagnosis**		
	**Yes**	21
	**No**	34
	**Unknown**	3
**Average Primary tumour size**		
	** < 10 cm**	19
	** ≥ 10 cm**	29
	**Unknown**	10

*TAH* Total Abdominal Hysterectomy

**Fig. 1:**
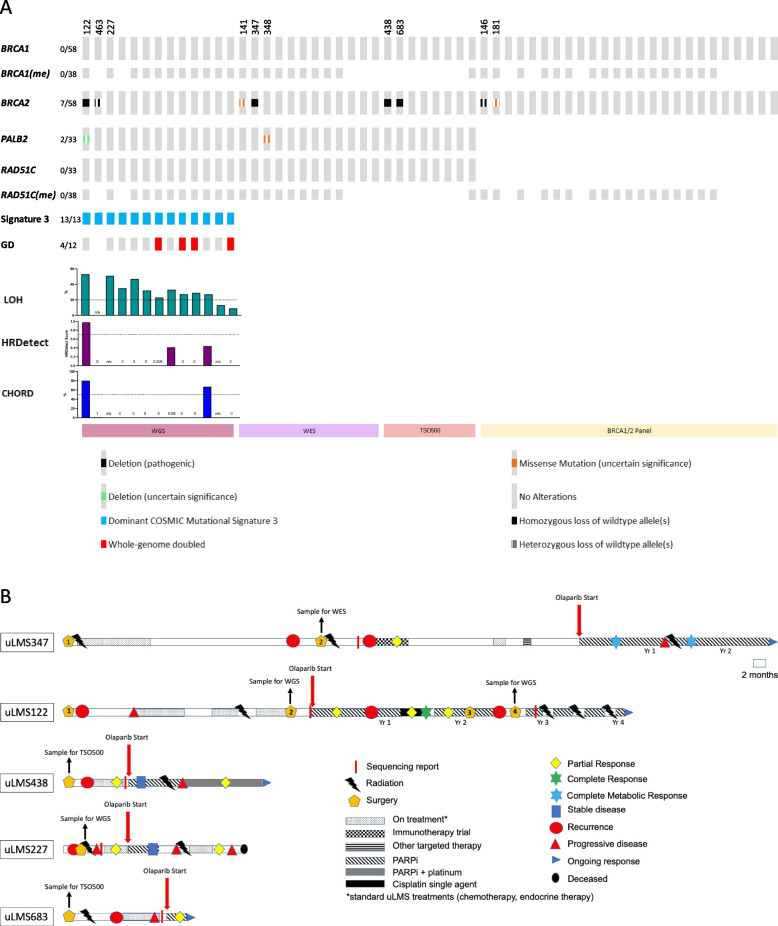
Molecular summary of HRD screening and clinical journey of the five HRD uLMS identified. **A** Summary of results from screening 58 uLMS samples via either whole genome sequencing (WGS), whole exome sequencing (WES), TruSight Oncology 500 (TSO500) panel testing or BRCA1/2 panel sequencing. Genome doubling (GD), percentage loss of heterozygosity (LOH) and results of MS-HRM methylation analysis of *BRCA1* and *RAD51C* promoters (denoted as BRCA1(me) and RAD51C(me), respectively) also shown. HRDetect scores and CHORD percentage readouts from UoM pipeline are as shown. Blanks indicate test was not run (for BRCA1(me) and RAD51C(me)), the tumour purity was not high enough to provide accurate scores or that the type of analysis used cannot report on the parameter. uLMS numbers refer to specific uLMS cases described in the text. **B** Timeline of uLMS patients who, based on HRD screening, received PARPi (olaparib) ± platinum therapy as part of their treatment history. Time of sample collection, type of molecular test, time of molecular reporting, and commencement of PARPi therapy are shown along with other types of therapy received and tumour responses where available

The results of HR pathway screening are shown in Fig. 1A with additional mutation detail provided in Supplementary Table 1. No germline mutations in any HR genes were detected in any of the cases assessed by WGS, WES or TSO500™. All 13 of the uLMS samples that underwent WGS had a dominant COSMIC mutational signature 3 (defined by the signature having a Mutational Pattern contribution value of >  = 3000 and ranked in the top three signatures by load). All but two had high genome-wide loss of heterozygosity (LOH) (> 0.2) and four cases were found to have whole genome doubling (wgd.test [51]), none of which were confirmed to be HRD. However, only two cases were found by WGS to harbour a pathogenic HRD gene mutation, both in *BRCA2* (a homozygous deletion in uLMS122; a heterozygous mutation in uLMS463). Only one case was designated as HRD by HRDetect [33] and CHORD [34] (uLMS122) and one case was characterised as HRD upon analysis of the resultant PDX (uLMS227, see below). WES was performed on 11 additional cases and detected a homozygous deletion in *BRCA2* in one case which also had a variant of unknown significance (VUS) in *BRCA2* (uLMS347). Two additional pathogenic HRD gene alterations (both homozygous deletions in *BRCA2*) were detected by tumour panel NGS testing (uLMS438, uLMS683). No mutations in *BRCA1* or *RAD51C/D* were detected in any sample, nor was hypermethylation of the *BRCA1* or *RAD51C* promoters detected (known to be important in HGSOC [53, 55]).

### PARP-inhibitor therapy improved patient outcomes in *BRCA2*-deleted uLMS

Of the four individuals found to have pathogenic alterations in *BRCA2* in their uLMS, all four received treatment with PARPi (Fig. 1B and Table 2). The first was an individual with uLMS122 harbouring a homozygous deletion in *BRCA2* who had an initial PR to treatment with olaparib, with a recurrence successfully treated with the addition of platinum chemotherapy to olaparib; and later upon the development of further minor progressive disease (PD), resulting in excision of a small lung nodule in which potential PARPi-resistance mutations in *PRKDC* were detected, received treatment with irradiation to two further minor PD nodules, and later gamma-knife stereotactic radiosurgery to multiple brain metastases, while intermittently continuing on single agent olaparib (ongoing), in metabolic CR (FDG-PET) (outside the brain), 49 months after starting olaparib (detailed below). The second individual with a *BRCA2* alteration in uLMS438 who received PARPi therapy, had biallelic loss of *BRCA2* detected in her uLMS via panel testing (TSO500™). Her uLMS progressed only two months after initial diagnosis, with lung metastases and she received first-line chemotherapy followed by single agent olaparib maintenance therapy. Imaging showed SD after two months of PARPi, then mixed response at four months. She received radiation to an enlarging pulmonary metastasis, but despite on-going olaparib developed PD within a further month. Single agent carboplatin was added seven months after starting PARPi and continued for 13 months (ongoing), with breaks required for haematotoxicity and increasing PR documented on serial scans. At the time of writing the second individual, uLMS438, continued in on-going PR, 19 months post-initiation of olaparib. The third individual, uLMS347, commenced olaparib as 5^th^ line therapy, > seven years after her original diagnosis. Partial response (close to a metabolic CR on FDG-PET) was achieved after five months of PARPi, followed by oligometastatic PD (intra-abdominal metastasis) a further eight months later, which was treated with radiotherapy; then continuation of olaparib, with a mediastinal metastasis appearing after 19 months total of PARPi-based therapy, with radiotherapy to the mediastinal metastasis planned, followed by continuation of olaparib. The fourth individual, uLMS683, experienced PD seven months after initial diagnosis and had a mixed response to 1^st^ line chemotherapy. Because of lung metastases she commenced olaparib with a partial metabolic response observed on FDG-PET after six weeks of PARPi. She remains in clinical response after 3 months of olaparib (ongoing) (Fig. 1B and Table 2).

**Table 2:** Outcomes of five individuals with uLMS treated with PARPi

Patient	Time on PARPi	OS^a^
^c^uLMS#122 olaparib; + cisplatin; ± RT	49 mo^b^	49 mo^b^
^d^uLMS#227 olaparib (ceased); chemotherapy	4 mo	9 mo
^c^uLMS#347 olaparib; RT	19 mo^b^	19 mo^b^
^c^uLMS#438 olaparib; + carboplatin	19 mo^b^	19 mo^b^
^c^uLMS#683 olaparib;	3 mo^b^	3 mo^b^

*mo* months

^a^since commencement of PARPi (olaparib) therapy

^b^continuing on therapy

^c^*BRCA2* deletion

^d^COSMIC signature 3

**Fig. 2:**
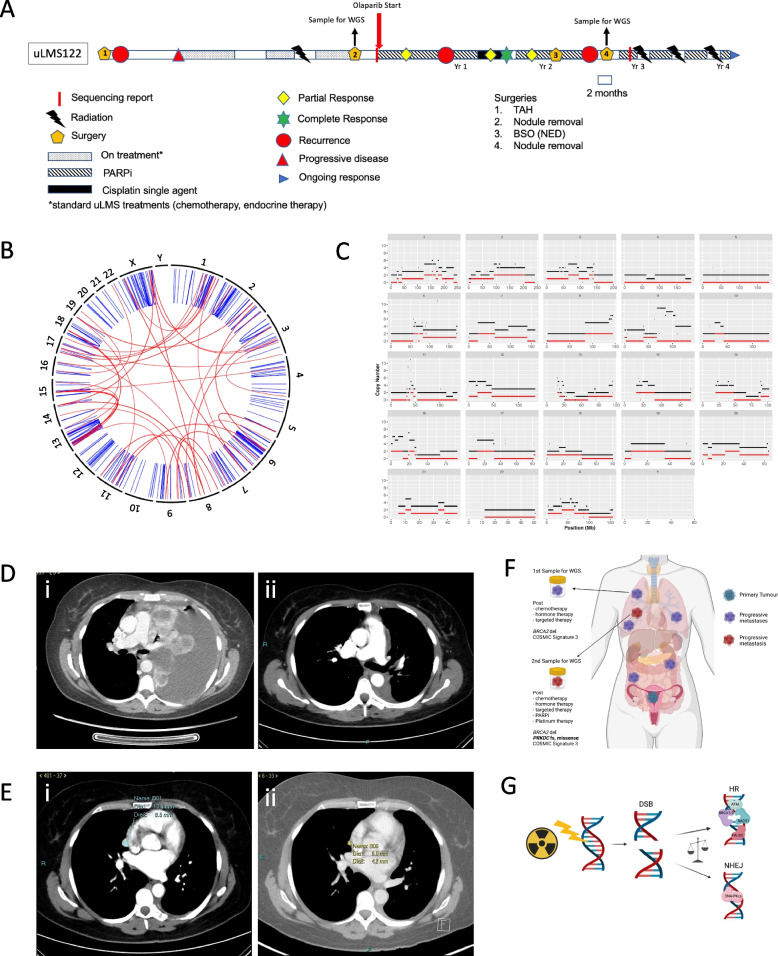
Patient with *BRCA2*-deleted, COSMIC signature 3 uLMS responded to PARPi. **A** Timeline of uLMS122 patient history (TAH = Total abdominal hysterectomy, BSO = Bilateral Salpingo-oophorectomy, NED = no evidence of disease). Repeated from Fig. 1B for ease of reference. **B** Structural variants plot generated from WGS data of first patient sample showing intra-chromosomal rearrangements. **C** Copy number profile generated from the first patient sample where total copy number is shown in black and minor copy number in red. Red at 0 indicates loss of heterozygosity. **D** Computerised tomography images of patient lungs at the point of recruitment to the SFRCP (i) and after 3 months of receiving olaparib (ii). **E** computerised tomography images of the patient lungs indicating a small recurrence (mediastinal nodule, blue cross) following the initial PARPi therapy (i) and following cisplatin plus PARPi. **F** Schematic of tumour samples analysed by WGS, with second sample showing additional *PRKDC* mutations (del = deletion, fs = frameshift). **G** Schematic depicting repair of DNA double stranded breaks (DSB) by either homologous recombination (HR) or Non-homologous end-joining (NHEJ), in which DNA PKcs plays a pivotal role

**Fig. 3:**
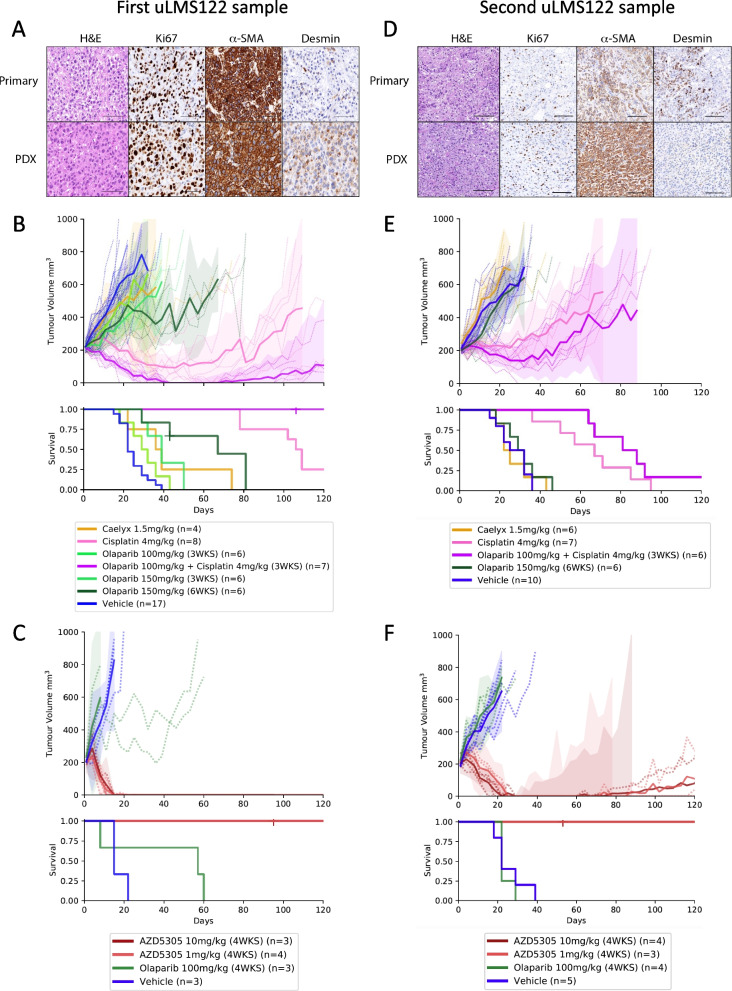
Two patient derived xenografts (PDX) generated from HRD uLMS tumour responsive to platinum therapy. **A** Immunohistochemistry panel showing concordant protein expression between first primary patient sample and PDX. H&E = Haematoxylin and Eosin, α-SMA = alpha smooth muscle actin. Scale bars represent 100 μm. **B** PDX tumour growth curves and survival in response to vehicle, standard therapy caelyx (pegylated doxorubicin; 1.5 mg/kg day 1, 8 and 18), olaparib (100 mg/kg or 150 mg/kg, daily Monday to Friday, 3 or 6 weeks), cisplatin (4 mg/kg day 1, 8 and 18) and the combination of olaparib (100 mg/kg daily Monday to Friday 3 weeks) and cisplatin (4 mg/kg day 1, 8 and 18). Data is shown as mean (solid lines, and individual tumours in dotted lines) with shaded areas representing 95% confidence intervals. **C** PDX tumour growth curves and survival in response to daily treatment for 4 weeks with vehicle, olaparib (100 mg/kg) or AZD5305 (1 mg/kg or 10 mg/kg). **D** Immunohistochemistry panel showing second PARPi resistant primary patient sample and PDX. H&E = Haematoxylin and Eosin, α-SMA = alpha smooth muscle actin. Scale bars represent 200 μm. **E** PDX tumour growth curves and survival in response to vehicle, caelyx (pegylated doxorubicin; 1.5 mg/kg day 1, 8 and 18), olaparib (150 mg/kg, daily Monday to Friday, 6 weeks) and cisplatin (4 mg/kg day 1, 8 and 18). Data is shown as mean with shaded areas representing 95% confidence intervals. **F** PDX tumour growth curves and survival in response to daily treatment for 4 weeks with vehicle, olaparib (100 mg/kg) or AZD5305 (1 mg/kg or 10 mg/kg)

The first individual referred to above, whose uLMS was designated uLMS122, was aged 45–49 years (hemi-decile) at diagnosis, when a uLMS positive for both estrogen and progesterone receptors (ER/PR) was detected after a total abdominal hysterectomy for suspected uterine fibroids. Pulmonary metastases were identified two months later. Hormonal therapy was ineffective and this was followed by doxorubicin resulting in PR after three cycles, ongoing response after six cycles, followed by a four-month treatment free interval (TFI). Docetaxel and gemcitabine were then commenced due to PD, with some response, and completion of six cycles. Three months later, despite irradiation to hilar lymph nodes, pazopanib was commenced for further PD, with no response. Ifosfamide was then delivered, resulting in severe toxicity. Dacarbazine was attempted, with impressive response and eight cycles were delivered followed by PD. At subsequent surgery for a symptomatic pleural effusion, with an Eastern Cooperative Oncology Group (ECOG) performance status of 3, a fresh pleural biopsy was obtained for urgent WGS, after having commenced five prior lines of therapy over 3.5 years (Fig. 2A). WGS detected a tumour mutational burden (TMB) of 2.9 mutations per Mega base; LOH of 0.48; homozygous deletion of *BRCA2*; and a dominant COSMIC mutational signature 3 (Figs. 1 and 2B,C), indicating that this uLMS was HRD. As a result, the patient was commenced on PARPi, olaparib, via a compassionate access program. The patient achieved a PR at four months (Fig. 2Di,ii), sustained until 12 months, before developing evidence of minor progression in a mediastinal lymph node (Fig. 2Ei). Because the majority of her uLMS was controlled on olaparib, and platinum chemotherapy had previously been shown to be safe in combination with olaparib in the clinic [56], cisplatin was added to the treatment schedule in an attempt not to lose control, in the context of heavy prior therapy, with no other good therapeutic options available. After three cycles of cisplatin and olaparib combination therapy, a PR was again observed. After six cycles of cisplatin in combination with olaparib, no appreciable activity was present on imaging (Fig. 3Eii), consistent with a CR. After two years on olaparib, bilateral salpingo-oophorectomy (BSO) was performed to determine whether a CR had been obtained, with low positive Fluoro-deoxy glucose positron emission tomography (PET) signal being observed in the adnexae, and upon analysis of the resected tissue no microscopic tumour was found, nearly six years following the original diagnosis.

Four months after proof of CR at six years post diagnosis, interval enlargement of a pulmonary nodule was noted and this was resected. WGS of this PARPi-resistant uLMS tissue confirmed the previously detected *BRCA2* deletion and CHORD and HRDetect analyses were consistent with the uLMS being HRD. Two heterozygous mutations were found in the *PRKDC* gene (Fig. 2F, Supplementary Table 1) that encodes DNA-PKcs, the catalytic subunit of DNA-dependent serine/threonine protein kinase (DNA-PK), in this PARPi-resistant sample. These *PRKDC* mutations were not detectable by WGS in the first patient sample. As DNA-PK is a core component of the classical non-homologous end-joining (c-NHEJ) pathway (Fig. 2G) and radiosensitivity of c-NHEJ deficient tumour cells has been observed [57, 58], the patient completed a course of stereotactic ablative radiotherapy to one additional chest wall nodule and concurrent radiation to a pelvic nodule, then continued with olaparib as maintenance therapy. Radiological CR was documented two months after this radiotherapy. After a further three months, multiple cerebral metastases were diagnosed and treated with gamma-knife stereotactic radiosurgery, with recommencement of olaparib and completion of weaning of corticosteroids a month later. After 49 months total of olaparib-based therapy, nearly eight years after her initial diagnosis, the individual remains in systemic CR, despite metastatic disease likely having been present at diagnosis.

### Patient-derived xenografts generated from HRD patient samples show concordance with patient responses to platinum therapy and PARPi

A PDX was generated from the initial pleural biopsy sample from uLMS122, which underwent WGS and enabled drug response to be assessed in vivo. IHC analysis was performed to confirm that the PDX matched the patient sample for major histomolecular characteristics (Fig. 3A). The PDX was treated with different doses and schedules of olaparib, as well as cisplatin which is our standard comparator drug in our PDX program for pre-clinical PARPi trials, and pegylated doxorubicin (caelyx) the standard therapy for advanced uLMS (Fig. 3B). Of the single agent olaparib schedules tested, treatment for six weeks with olaparib at 150 mg/kg provided the best response (Table 3). However, greater response was observed for single agent cisplatin, a drug not typically used as standard of care treatment of uLMS. The use of cisplatin together with olaparib, 100 mg/kg, resulted in a deeper and more sustained response, with a CR (tumour volume smaller than 30mm^3^ for three consecutive weeks) observed in 7/7 tumours, compared to just 1/8 tumours in the cisplatin alone treatment group. No reduction in tumour volume was observed in response to treatment with pegylated doxorubicin.

**Table 3:** Responses of first uLMS122 Patient Derived Xenograft to PARPi and standard therapy in vivo

	**Test Statistic**^a^	***P*-value**
Vehicle vs caelyx 1.5 mg/kg	4.76	0.03
Vehicle vs olaparib 100 mg/kg	2.40	0.1
Vehicle vs olaparib 150 mg/kg (3WKS)	9.54	0.002
Vehicle vs olaparib 150 mg/kg (6WKS)	13.64	0.0002
Vehicle vs cisplatin 4 mg/kg	21.33	0.00000
Vehicle vs olaparib 100 mg/kg + cisplatin 4 mg/kg	19.20	0.00001
Cisplatin vs olaparib 100 mg/kg + cisplatin 4 mg/kg	8.63	0.003
Vehicle vs AZD5305 10 mg/kg	5.21	0.02
Vehicle vs AZD5305 1 mg/kg	6.64	0.01
Olaparib 100 mg/kg (4WKS) vs AZD5305 10 mg/kg	5.05	0.02
Olaparib 100 mg/kg (4WKS) vs AZD5305 1 mg/kg	6.62	0.01

^a^Pairwise Log Rank Tests used to test the null hypothesis

A PDX was also generated from the second sample from this patient, which was found by WGS to contain two heterozygous mutations in the *PRKDC* gene encoding DNA-PKcs, the catalytic subunit of DNA-PK, which were not present in WGS performed on the first patient sample. Almost all other major histomolecular characteristics were retained in the second PDX (Fig. 3D). As in the patient, this second PDX was resistant to olaparib, but remained responsive to cisplatin, although less responsive than the PDX from the earlier uLMS122 sample (cisplatin median time to harvest 67 days vs 109 days; Fig. 3E, Table 4). The highly selective, potent PARP1 inhibitor and PARP1 − DNA trapper, AZD5305, is now in the clinic via the PETRA trial (NCT04644068) [59]. Strikingly, both PDX derived from this case, demonstrated early, deep responses to the more potent PARP1-specific PARPi, AZD5305 [59, 60] (Fig. 3C and F, Tables 3 and 4), despite the fact that the PDX from the second sample was resistant to treatment with olaparib (150 mg/kg for 6 weeks, Fig. 3E, Table 4). The first PDX demonstrated sustained CR for 120d (end of experiment). The second PDX also showed rapid CR to 80d, despite containing *PRKDC* mutations, known to confer resistance to PARPi [61, 62]. In contrast, all mice treated with single-agent olaparib underwent progression regardless of which PDX (uLMS122 PDX1 time to PD 7 days for vehicle vs 7 days olaparib vs > 120 days AZD5305; *p* value for olaparib vs AZD5305 (1 mg/kg) = 0.01; uLMS122 PDX2 time to PD 7 days for vehicle vs 7 days olaparib vs > 120 days AZD5305; *p* value for olaparib vs AZD5305 (1 mg/kg) = 0.01).

**Table 4:** Responses of second uLMS122 Patient Derived Xenograft to PARPi and standard therapy in vivo

	**Test Statistic**^a^	***P*-value**
Vehicle vs caelyx 1.5 mg/kg	0.015	0.9
Vehicle vs olaparib 150 mg/kg (6WKS)	0.65	0.4
Vehicle vs cisplatin 4 mg/kg	13.37	0.0003
Vehicle vs olaparib 100 mg/kg + cisplatin 4 mg/kg	13.26	0.0003
Cisplatin vs olaparib 100 mg/kg + cisplatin 4 mg/kg	1.40	0.2
Vehicle vs AZD5305 10 mg/kg	8.01	0.005
Vehicle vs AZD5305 1 mg/kg	6.23	0.01
Olaparib 100 mg/kg (4WKS) vs AZD5305 10 mg/kg	7.60	0.006
Olaparib 100 mg/kg (4WKS) vs AZD5305 1 mg/kg	6.12	0.01

^a^Pairwise Log Rank Tests used to test the null hypothesis

### HRD signature and copy-number analysis identifies additional patients with uLMS who may potentially benefit from PARPi

**Fig. 4:**
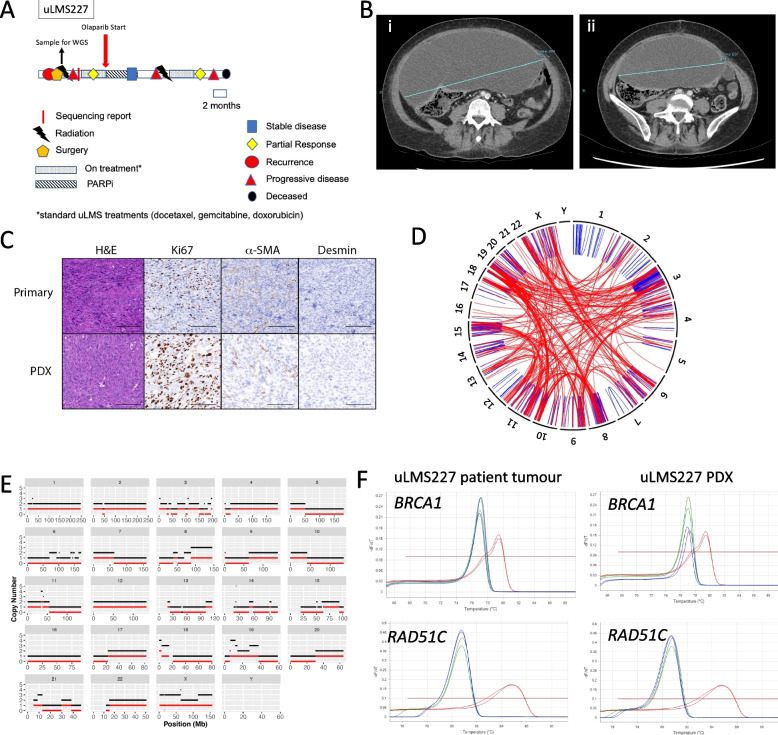
Patient with HRD uLMS with no HR pathway gene mutations response to PARPi. **A** Timeline of uLMS227 patient history. Repeated from Fig. 1B for ease of reference. **B** computerised tomography images of the patient abdomen prior to (i) and 3 months after initiation of PARPi therapy (ii). **C** Immunohistochemistry panel showing concordant protein expression between primary patient sample and PDX. H&E = Haematoxylin and Eosin, α-SMA = alpha smooth muscle actin. Scale bars represent 200 μm. **D** Structural variants plot generated from WGS data of the uLMS227 PDX sample showing intra-chromosomal rearrangements. **E** Copy number profile generated from the uLMS227 PDX sample where total CN is shown in black and minor CN in red. **F** Methylation analysis of the *BRCA1* and *RAD51C* promoters. Red lines indicate a 100% methylated control, blue lines indicated 0% methylated control and the green line represents the uLMS227 sample (both patient and PDX sample)

The patient whose uLMS was designated uLMS227, was diagnosed with FIGO Stage I, poorly differentiated ER/PR negative uLMS, aged between 50–55 years and was chemotherapy-naïve when tissue was obtained from the primary tumour at the time of initial surgery and then prepared for WGS (Fig. 4A). Despite anatomical pathology review reporting 90% tumour purity in the sample, analysis of the WGS data revealed that the sampled uLMS was of low cellularity (< 30%), potentially attributable to poor fixation of the primary sample or heterogeneity of cell types within uLMS, but providing limited sensitivity of WGS for mutation detection. Nevertheless, a dominant COSMIC mutational signature 3 (Supplementary Fig. 1) was identified, although CHORD and HRDetect analyses could not be performed due to poor tumour purity. No pathogenic alterations in HRD genes were observed to account for the dominant signature 3. On completion of the chosen standard first-line therapy, the patient was commenced on maintenance olaparib via a compassionate access program.

At four months post initiation of olaparib, a minor response was observed consistent with stable disease on CT scan (Fig. 4B). However, at that time, the development of erythroid hypoplasia necessitated cessation of olaparib, following which progressive disease was detected after four months. The patient subsequently received second-line therapy and a CT scan performed one month after completion of four cycles showed a reduction in all metastatic sites. Unfortunately, this initial response was followed by rapid PD and the patient died some months later.

In order to better determine the HRD status of uLMS227, we analysed the PDX which had been generated from the primary uLMS227 sample. This PDX uLMS227 showed IHC staining consistent with the original tumour (Fig. 4C). WGS analysis of the PDX uLMS227 sample confirmed the presence of the dominant COSMIC mutational signature 3 (Fig. 4D). The greater purity of the PDX sample allowed identification of a loss-of-heterozygosity (LOH) copy number profile characteristic of HRD malignancies (Fig. 4E) in keeping with the COSMIC mutational signature 3. Interestingly, despite this very clear PDX LOH CN profile, no mutations in any HR related genes were detected. Methylation analysis of the *BRCA1* and *RAD51C* promoters was performed on both the patient and PDX uLMS227 samples, to further investigate the cause of the COSMIC mutational signature 3. Neither promoter was methylated in either the baseline patient or PDX samples (Fig. 4F), as had been observed, as expected, for the *BRCA2*-deleted uLMS122 samples (Supplementary Fig. 2)).

### HR Proficient uLMS PDX shows no response to PARPi

**Fig. 5:**
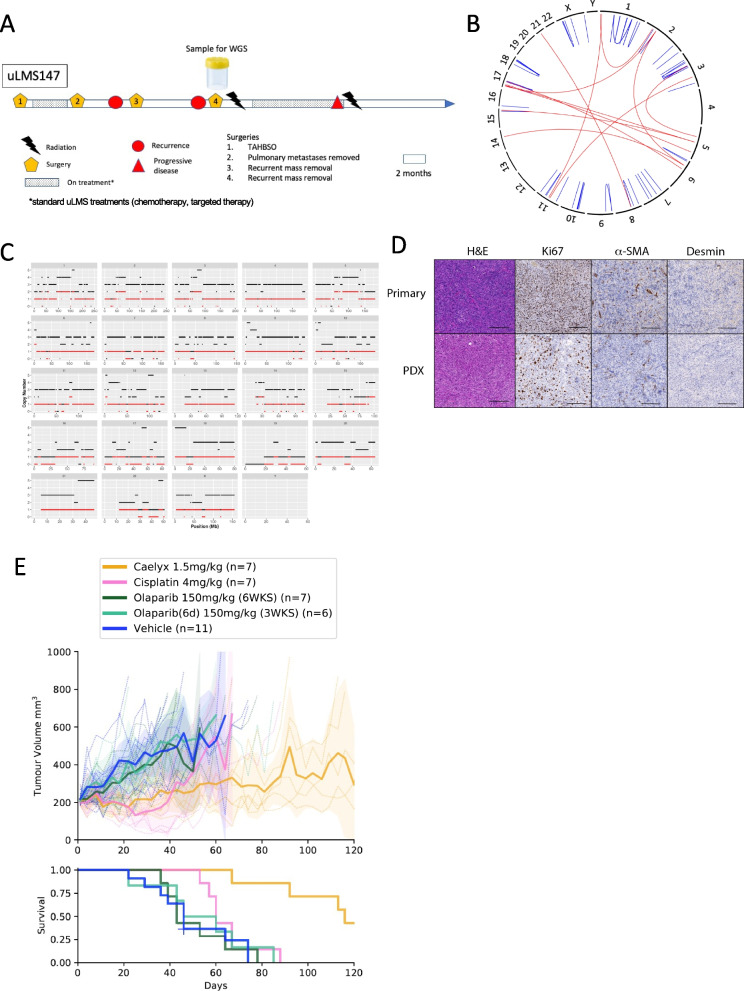
HRP uLMS PDX does not respond to PARPi. **A** Timeline of uLMS147 patient history. **B** CIRCOS plot generated from patient WGS data showing intra-chromosomal rearrangements, and (**C**) Copy number profile, where total CN is shown in black and minor CN in red. **D** Immunohistochemistry panel showing concordant protein expression between primary patient sample and PDX. H&E = Haematoxylin and Eosin, α-SMA = alpha smooth muscle actin. Scale bars represent 200 μm. **E** PDX tumour growth curves and survival on treatment with vehicle, caelyx (pegylated doxorubicin; 1.5 mg/kg 1, 8 and 18), olaparib (150 mg/kg, daily Monday to Friday or Monday to Sat, 6 weeks) and cisplatin (4 mg/kg day 1, 8 and 18). Data is shown as mean with shaded areas representing 95% confidence intervals

In contrast, the patient whose uLMS was designated uLMS147, was diagnosed with metastatic FIGO stage IV dedifferentiated uLMS under the age of 50 years (Fig. 5A). She was treated with one line of systemic therapy and required several surgical resections of metastatic disease before WGS was performed on a sample of her uLMS. Similar to uLMS227, the estimated tumour purity contained within the biopsy sample was too low (< 30%) for somatic variant analysis using the UoM pipeline, including HRDetect or CHORD analyses. Using the WEHI pipeline, no mutations in any of the HR pathway genes were detected, and there was no evidence of the LOH pattern typical of HRD tumours (LOH 0.13) (Fig. 5B, C) and therefore this sample was characterised as being HRP.

A PDX derived from uLMS147 showed similar IHC staining to the original tumour (Fig. 5D). There was a significant reduction in tumour growth in response to pegylated doxorubicin (caelyx). Unlike uLMS122, however, the PDX from uLMS147 was unresponsive to both three and six weeks of olaparib at 150 mg/kg (Fig. 5D, Table 5). There was an initial response to cisplatin, with inhibition of tumour growth observed during the treatment period and extending to 40 days, but ultimately the PDX uLMS147 was resistant to cisplatin.

**Table 5:** Responses of uLMS147 Patient Derived Xenograft to PARPi and standard therapy in vivo

	**Test Statistic**^a^	***P*-value**
Vehicle vs caelyx 1.5 mg/kg	11.61	0.0007
Vehicle vs olaparib (6d) 150 mg/kg (3WKS)	0.17	0.7
Vehicle vs olaparib 150 mg/kg (6WKS)	0.017	0.9
Vehicle vs cisplatin 4 mg/kg	1.46	0.2

^a^Pairwise Log Rank Tests used to test the null hypothesis

## Discussion

Durable benefit with PARPi has been reported for individuals with *BRCA2*-mutated uLMS [15, 63], with one prior CR being documented in response to PARPi [15]. In addition, three individuals with uLMS with homozygous *BRCA2-*deletion received benefit from treatment for > 14 months (ongoing response), including one CR and two PRs, with the combination of PARPi (talazoparib) and the anti-PD-L1i, nivolumab, in the JAVELIN BRCA/ATM phase 1b clinical trial [64]. Despite these observations, screening for mutations in *BRCA1/2* and the HR pathway is not routinely performed in the clinic for uLMS despite the lack of available alternative efficacious therapeutic options. In Australia, reimbursed molecular sequencing is available for individuals with sarcoma (including uLMS) for 21 specified genes (none of which are in the HR pathway) and the current ad hoc testing available through research studies and clinical trials is insufficient to identify all uLMS patients who would benefit from PARPi access.

This study provides real-world evidence of HRD in uLMS cases and one example of profound, life-changing clinical benefit for an individual with uLMS122 treated with olaparib for 3.5 years, requiring a course of cisplatin combined with olaparib after one year on olaparib and later on, successful short courses of radiotherapy in an oligometastatic setting. We showed that the addition of cisplatin to olaparib resulted in CR of this *BRCA2* homozygously deleted uLMS in an individual who had been heavily pre-treated. We also report an ongoing PR in a patient with uLMS347 harbouring a biallelic loss of *BRCA2*, again requiring olaparib/platinum combination therapy, where single agent olaparib was insufficient for control. In addition, we showed potential benefit for two other individuals, who also accessed olaparib treatment following the detection of HRD, as evidenced by *BRCA2* homozygous deletion in uLMS348 (> 13 months with olaparib-based therapy (plus radiation) and Signature 3 in uLMS227, using a comprehensive molecular screening approach. Due to poor tumour purity, HRD was ultimately confirmed by LOH copy number profile in the resulting high tumour-purity uLMS PDX 227, as was the absence of any HRD gene mutation or promoter methylation to account for the HRD. Thus, we identified that COSMIC mutational signature 3, in addition to a high level of genome wide LOH, may also indicate susceptibility to PARPi therapy in uLMS even in the absence of a detected mutation in HRD pathway genes. This supports the need for coordinated research screening of these patients by WGS if no HRD aberrations are found by BRCA/panel analysis. Together, our work extends previous findings [10, 14, 15, 28, 29] and highlights a pressing need to identify subsets of individuals with uLMS who may respond to targeted therapy in this rare cancer type. Individuals with uLMS have a paucity of treatments currently available for their extremely aggressive cancer and stand to benefit from a readily available oral therapy, PARPi, with quite transformational responses.

### Agents aiding PARPi response in uLMS

The favourable response of PDX uLMS122 to cisplatin was atypical for uLMS but consistent with previously demonstrated platinum sensitivity observed for a range of gynaecological cancers with HRD [65]. There is mixed low-level evidence for efficacy of cisplatin-based combination therapies in sarcomas. Cisplatin plus pemetrexed therapy was associated with sustained responses in patients with advanced and refractory soft tissue sarcoma (STS) [66], and cisplatin-based chemotherapy resulted in a rapid major partial response in a patient with BRCA2-deficient STS [67]. In addition, in an HRD setting, there is rationale for using platinum-based therapies in sarcoma, based on HRD PARPi literature, where platinum followed by PARPi maintenance approach has been used, with profound success. Single-agent cisplatin was initially employed to treat uLMS, but small cohort studies in the late 1980s and 1990s showed little or no response [68, 69]. Other chemotherapy combinations, including with cisplatin, have been tested in uLMS, but with limited success [70, 71]. It is possible that if these prior studies had first screened patients for HRD and stratified their cohort accordingly, the outcomes to platinum for HRD uLMS patients may have been more favourable. Notably, a recent clinical trial combining a different DNA alkylating agent, temozolomide, with olaparib opened in 2019 (NCT03880019) due to pre-clinical evidence of the efficacy of this combination in uLMS cell lines [72]. This study reported that 23% of patients achieved an objective response within six months of initiating combination olaparib/temozolomide therapy [73]. We await the translational analysis of this trial, but as we and others have showed more conservative proportions of individuals to have HRD in their uLMS (~ 10%), the higher RR observed in the combination olaparib/temozolomide trial of 22% could indicate that strictly defined HRD (~ 10% in our and other’s series) may not solely underpin responses to PARPi/temozolomide or perhaps to other PARPi combination regimens, such as PARPi/cisplatin. Our findings highlight the potential importance of adaptive approaches for future PARPi trial designs in this rare disease.

### Mechanisms of resistance to PARPi and a matched approach to surmount it in uLMS

The CR achieved by patient uLMS122 in response to cisplatin/olaparib combination therapy was intriguing and in keeping with known responses of other breast and gynaecological cancers to platinum/PARPi regimens [20, 74]. Nevertheless, a recurrence in this patient did occur, allowing for subsequent WGS on the PARPi resistant lung nodule. The *PRKDC* mutations detected in this recurrent sample is to our knowledge the first such description in uLMS. The *PRKDC* gene encodes DNA-PKcs, a key protein in the c-NHEJ pathway, which competes with the HR pathway for DNA double strand break repair during S and G2 phases of cell cycle [61, 75]. The c-NHEJ repair pathway does not rely on homology like HR, this mode of repair can occur at any phase of cell cycle and may become abortive/error-prone in the absence of HR repair, creating small DNA-damaging deletions [61, 75]. Loss or inhibition of core c-NHEJ components, including DNA-PKcs, has been described as a mechanism of PARPi resistance in *BRCA2*-mutant ovarian cancer cells previously [61, 62]. Therefore, we propose that deregulated c-NHEJ was the mechanism of PARPi resistance most likely responsible for the development of the resistant lung nodule, given that secondary mutations in *BRCA2* would not be likely to revert a *BRCA2* deletion to wild-type [76] and, as expected, secondary mutations in *BRCA2* were not observed upon WGS analysis of this case, following progression on PARPi. Fortunately, cells with decreased or absent c-NHEJ are particularly radiosensitive [57, 58], making local radiotherapy a practical targeted therapeutic option for this individual who had only low volume disease recurrence, followed by continuation of single agent olaparib therapy in this oligo-metastatic setting [77]. Indeed, an additional subsequent approach is to determine whether a more potent and less toxic PARPi might be even more effective in the clinic than the current PARPi, abrogating the need for combination therapies. The highly selective, potent PARP1 inhibitor and PARP1 − DNA trapper, AZD5305 [59] caused early, deep responses in both uLMS122 PDX, despite the fact that the PDX from the second sample was resistant to treatment with olaparib and contained two *PRKDC* mutations, known to confer resistance to PARPi [61, 62]. It would be timely to consider including individuals with HRD uLMS in trials of new PARPi such as in the PETRA trial (NCT04644068) [59] or for individuals with cerebral metastases, trials of AZD9574, a novel, brain penetrant PARP-1 selective inhibitor [78].

### PARPi-induced erythroid toxicity in uLMS

For uLMS227, despite intolerance to olaparib therapy in the clinic after five months, due to haematologic toxicity, the individual’s uLMS responded to subsequent chemotherapy, possibly in keeping with prolonged responses observed for time to subsequent therapies following prior PARPi in ovarian cancer (for example, [79]). However, with such a rare cancer type, and the paucity of information available for duration of responses to second-line therapies in uLMS, it is not possible to be certain, whether or not olaparib provided a benefit (nine-month interval between 1^st^ line and 2^nd^ line chemotherapy regimens, including the time on olaparib therapy (five of the nine months)). Anaemia and neutropenia are common adverse events in trials of both PARPi single agent and PARPi/platinum combination therapies [80–83]. Efforts to minimise haematological toxicities of PARPi are underway. PARP-2 has been demonstrated to have an important role in sustaining erythropoiesis [84], and development of more targeted PARPi such as AZD5305 with enhanced PARP1 selectivity has been shown to have reduced haematologic toxicity in a pre-clinical model [60]. Combining PARPi therapy with a chemo-protector, such as a CHK2 inhibitor, has also been proposed, with a pre-clinical study demonstrating prevention of cytotoxicity in B cells by the CHK2 inhibitor, BML-277, chosen as a result of a CRISPR/cas9 genetic screen [85].

### HRD screening approaches in uLMS

As demonstrated here, an appropriate screening strategy for detection of HRD must be carefully considered. Panel tests are relatively inexpensive with a rapid turn-around time requiring relatively low tumour purity. However, screening for mutations in common HRD genes (*BRCA1, BRCA2, PALB2, RAD51C*) may not be sufficient to detect all cases of HRD in uLMS. We have used a combination of both COSMIC mutational signature 3 dominance and high genome wide LOH to designate a uLMS as being HRD, in the absence of an HRD gene mutation, such as in the case for uLMS227. This was subsequently supported by the greater purity of the PDX uLMS227 sample enabling observation of an LOH copy number profile which was characteristic of HRD. WGS can detect mutational signatures whilst both WGS and WES can provide LOH patterns. Given that the COSMIC signatures were trained on data sets comprising more common cancer types, it may be appropriate for caution to be applied when interpreting mutational signature results for rare cancers, at least for uLMS. Indeed, Choi and colleagues also identified a higher than expected frequency (25%) of dominant COSMIC mutational signature 3 in their cohort of uLMS [14]. Algorithmic assessment of HRD, for example using CHORD [34] and HRDetect [33] may also require further validation in certain rare cancer subtypes.

The timing of screening is also critical, as evidenced in the cases of uLMS122 and uLMS347. These individuals had received three-five beneficial lines of prior cytotoxic/other therapies during the first three years of their disease, with increasingly rapid relapses. If the *BRCA2* deletions identified in these individuals had been detected at primary diagnosis, their quality of life would likely have been improved earlier, by being considered for PARPi therapy up to three-seven years earlier. It is astounding that despite being heavily pre-treated, these individuals still received considerable benefit from olaparib, suggesting that more efficacious approaches (combinations or more potent PARPi, or both) could be even more successful.

As a result of our findings, we recommend BRCA panel testing be performed on FFPE for all cases of uLMS, aiming for early detection of HRD with subsequent early access to PARPi. *BRCA2* is the major recurring defective HRD gene in this disease and hence analysis of *BRCA2* is most important. Due to tumour heterogeneity within the specimen, we found it helpful to request two blocks in case of assay failure. The rationale for requesting two different FFPE blocks concurrently included preventing delays (inherent with having to make a second request), the result could have a profound impact on the individual’s outcome, there was usually a large amount of tumour tissue available and we confirmed that we would return both blocks within a reasonable timeframe. We also advocate for the collection of fresh tumour tissue at primary diagnosis of uLMS, whenever possible, to enable a comprehensive approach to molecular screening: if no HRD abnormality is detected on panel testing, then WGS or high-quality WES (less expensive than WGS, but more reliable analysis on fresh tissue than when performed on FFPE) should be performed, or if only FFPE tumour is available then WES could be considered, until methods improve and costs reduce such that WGS is feasible on FFPE. Whilst WES/WGS is not possible in all centres, tissue collection and analysis could be coordinated by a relevant centralised research program such as is performed by the WEHI-Stafford Fox Rare Cancer Program in Australia. By developing this uLMS HRD analysis process to be the gold standard approach for personalised therapy, such analyses could be transformative for individuals with uLMS found to have HRD early in their disease journey. Prolonged PR of greater than one year, or even CR such as reported in this study and by Hensley et al. [15] and in the JAVELIN BRCA/ATM trial [64] could enable improved quality of life and may allow prolongation of life. Importantly, Signature 3/LOH analyses should be included in the translational analysis of individuals with uLMS in clinical trials, in order to identify those individuals who will most likely benefit from inclusion in subsequent clinical trials using PARPi combination therapies. The newer PARP1-specific PARPi may also have an important role in the treatment of uLMS and consideration should be given to including cohorts of uLMS patients in existing PARPi umbrella trials of newer PARPi agents. Routine identification of HRD lesions in uLMS will enable individuals with a uLMS that is unlikely to respond to PARPi combination therapies, to receive more appropriate therapy, including other clinical trials.

## Conclusions

There is a paucity of effective therapies currently available for individuals with uLMS, a rare gynaecological cancer. In this study we show that a national approach for screening for HRD in uLMS identified 5/58 (9%) cases to have HRD, helping to transform the lives of those individuals with HRD uLMS, as responses to PARPi-containing therapeutic regimens, including the combination of PARPi/platinum or PARPi/RT, can be long-lasting and well tolerated.

## Supplementary Information

**Additional file 1: Supplementary Table 1. **HRD mutations identified by screening uLMS from 58 individuals.

**Additional file 2: Supplementary Figure 1.** (A) Structural variants plot generated from WGS data of uLMS227 primary patient sample showing intra-chromosomal rearrangements. (B) Copy number profile generated from the uLMS227 patient sample. Total copy number is shown in black and minor copy number in red. **Supplementary Figure 2. **Methylation analysis of the *BRCA1 *and *RAD51C *promoters. Red lines indicate a 100% methylated control, blue lines indicated 0% methylated control and the green line represents the uLMS122 sample (both patient and PDX sample).

## Data Availability

The datasets generated and analysed during the current study are available from the corresponding author on reasonable request.

## Abbreviations

CHORD: Classifier of homologous recombination
c-NHEJ: Classical non-homologous end-joining
COSMIC: Catalogue of somatic mutations in cancer
CR: Complete response
HGSOC: High grade serous ovarian carcinoma
HR: Homologous recombination
HRD: Homologous recombination deficient
HRP: Homologous recombination proficient
LOH: Loss of heterozygosity
NGS: Next generation sequencing
PARPi: Poly (ADP-ribose) polymerase inhibitor
PD: Progressive disease
PDX: Patient derived xenograft
PFS: Progression free survival
PR: Partial response
SD: Stable disease
uLMS: Uterine leiomyosarcoma
WES: Whole exome sequencing
WGS: Whole genome sequencing
