# Supplementary material for: Targeting homologous recombination deficiency in uterine leiomyosarcoma
Source: J Exp Clin Cancer Res. 2023 May 4;42:112. doi: 10.1186/s13046-023-02687-0 (PMC10157936; doi:10.1186/s13046-023-02687-0)
Supplement: Supplementary file 1 — Additional file 1: Supplementary Table 1. HRD mutations identified by screening uLMS from 58 individuals. [file 13046_2023_2687_MOESM1_ESM.pdf]

Dall et al. Supplementary Table 1

Sample ID	HR status of uLMS	Gene	Variant	Classification (ClinVar)	Loss of wildtype allele(s)	CHORD	HRDetect	Method of Detection
uLMS#122[1]	**HRD**	BRCA2	deletion	N/A	homozygous	72%	0.906	WGS
uLMS#122[1]	**HRD**	PALB2	deletion	N/A	heterozygous			WGS
uLMS#122[2]	**HRD**	PRKDC	NM_006904:c.4778T>C: exon37:p.V1593A	Uncertain	N/A	80%	0.979	WGS
uLMS#122[2]	**HRD**	PRKDC	NM_006904:c.2476_2503del:exon22:p.F826fs	Uncertain	N/A			WGS
uLMS#227	**HRD**		COSMIC mutational signature 3	N/A		N/A	N/A	WGS
uLMS#347^	**HRD**	BRCA2	NM_000059.3: c.464G>C	Uncertain	Uncertain	N/A	N/A	WES
uLMS#347^	**HRD**	BRCA2	deletion	N/A	homozygous	N/A	N/A	WES
uLMS#438	**HRD**	BRCA2	deletion	N/A	homozygous	N/A	N/A	TSO500
uLMS#683	**HRD**	BRCA2	deletion	N/A	homozygous	N/A	N/A	TSO500
uLMS#463	HRP	BRCA2	NM_000059.3:c.1953_1957dupTTCTG:p.E653fs	Pathogenic	heterozygous	1%	0	WGS/BRCA1/2 Panel
uLMS#146	HRP	BRCA2	NM_000059.3:c.1_10257del	Pathogenic	heterozygous	N/A	N/A	BRCA1/2 Panel

[1] First Sample analysed
[2] Second Sample analysed
^ Germline variant
