# Supplementary material for: Targeting homologous recombination deficiency in uterine leiomyosarcoma
Source: J Exp Clin Cancer Res. 2023 May 4;42:112. doi: 10.1186/s13046-023-02687-0 (PMC10157936; doi:10.1186/s13046-023-02687-0)
Supplement: Supplementary file 2 — Additional file 2: Supplementary Figure 1. (A) Structural variants plot generated from WGS data of uLMS227 primary patient sample showing intra-chromosomal rearrangements. (B) Copy number profile generated from the uLMS227 patient sample. Total copy number is shown in black and minor copy number in red. Supplementary Figure 2. Methylation analysis of the BRCA1 and RAD51C promoters. Red lines indicate a 100% methylated control, blue lines indicated 0% methylated control and the green line represents the uLMS122 sample (both patient and PDX sample). [file 13046_2023_2687_MOESM2_ESM.pdf]

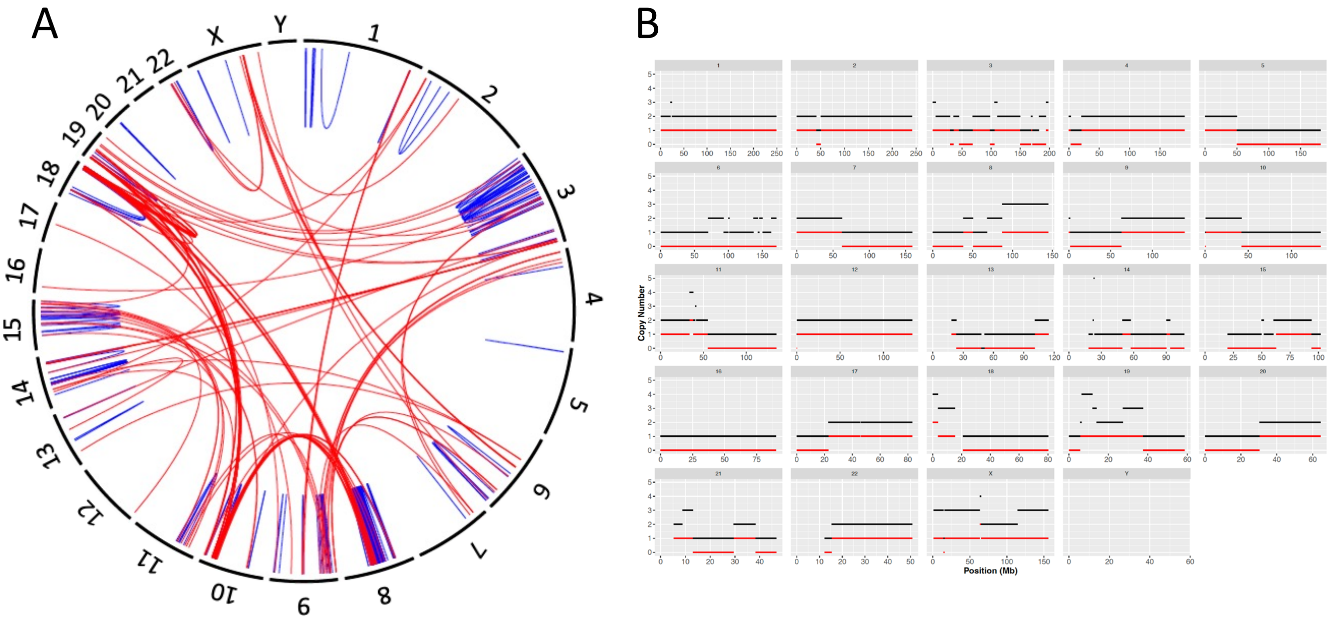

Supplementary Figure 1. (A) Structural variants plot generated from WGS data of uLMS227 primary patient sample showing intra-chromosomal rearrangements. (B) Copy number profile generated from the uLMS227 patient sample. Total copy number is shown in black and minor copy number in red.

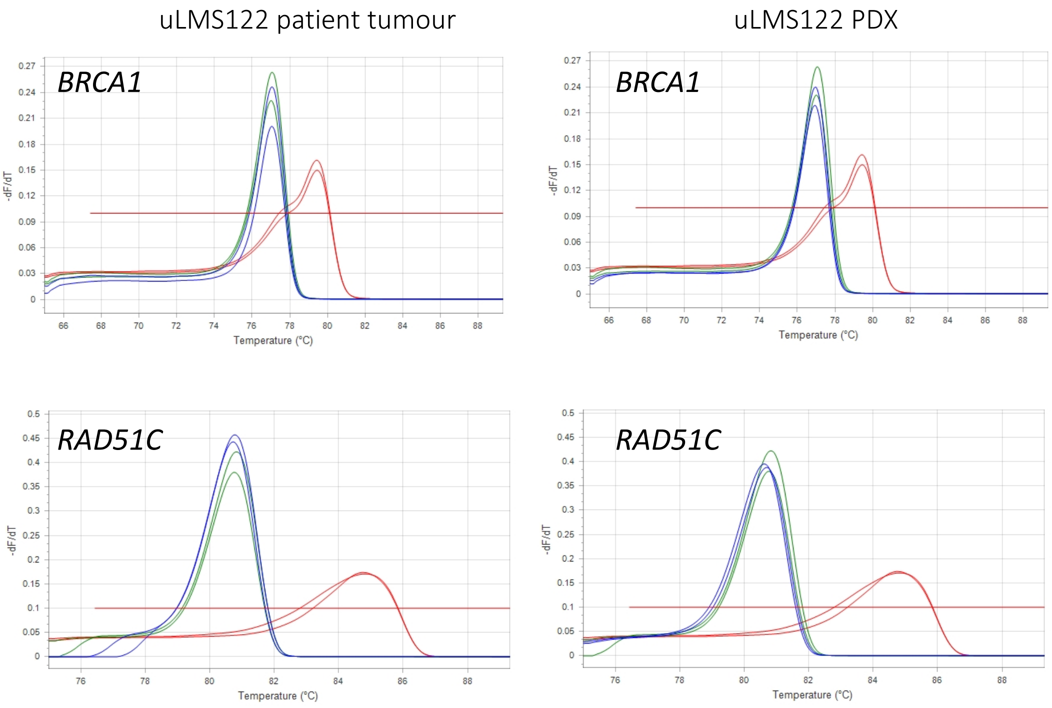

Supplementary Figure 2. Methylation analysis of the *BRCA1* and *RAD51C* promoters. Red lines indicate a 100% methylated control, blue lines indicated 0% methylated control and the green line represents the uLMS122 sample (both patient and PDX sample).
